# Impact of the magnetic field‐assisted freezing on the moisture content, water migration degree, microstructure, fractal dimension, and the quality of the frozen tilapia

**DOI:** 10.1002/fsn3.2653

**Published:** 2021-11-07

**Authors:** Heyun Wei, Kaixuan Luo, Renhao Fu, Xiangdong Lin, Aiguo Feng

**Affiliations:** ^1^ School of Food Science and Engineering Hainan University Haikou China; ^2^ Collaborative Innovation Center of Provincial and Ministerial Co‐construction for Marine Food Deep Processing Dalian Polytechnic University Dalian China

**Keywords:** low‐field nuclear magnetic resonance, low‐temperature freezing, tilapia, water migration

## Abstract

In this study, we determined the effect of a magnetic field applied during refrigeration in improving the quality of frozen tilapia. Alternating magnetic fields of 10 G, 20 G, 30 G, 40 G, and 50 G were applied during a low‐temperature freezing treatment on the back, abdomen, and tail of tilapia. The control group was set at 0 G. A correlation analysis for the fish films after treating with different magnetic field strengths was carried out. The results showed that when the magnetic field was applied to assist freezing, the frozen quality of the tilapia was significantly improved, and the water separation and residual damage were reduced. The felled muscle tissue decreased, the fractal dimension value increased, the hardness decreased, and the elasticity increased. However, the impact of the magnetic field on the quality of the frozen tilapia did not change with an increase in the magnetic field strength. The effect on the back samples was more prominent when the fish were exposed to the magnetic field strength of 40 or 50 G. A magnetic field strength of 50 G was the most effective for the abdominal and tail samples. However, no significant difference was observed in the groups exposed to 10 and 20 G of magnetic fields.

## Graphical abstract

The application of alternating magnetic field‐assisted freezing in tilapia can be helpful in tilapia storage. The effect of alternating magnetic fields on different parts of frozen tilapia can provide a theoretical basis for tilapia quality research. The experimental results of alternating magnetic field‐assisted freezing in tilapia can provide a theoretical basis for the application of the magnetic field.

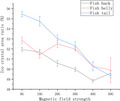

## 1 INTRODUCTION

Tilapia, also known as the African crucian carp, is a freshwater farmed fish cultivated worldwide by the aquaculture industry. It has high economic value, and its data are supported by key scientific research. China is the world's largest tilapia farming and supplying country (China Agriculture Press, 2020). Magnetic resonance imaging technology refers to the use of nuclear magnetic resonance principles. Through spatial coding technology, the radio frequency signal emitted from hydrogen atoms is processed by a computer to form an image. This technology has been widely used in food and agriculture, petroleum surveying, porous media, polymer materials, and other fields and has become an increasingly popular scientific research method (Zhao et al., 2017). Cui Zhiyong analyzed the relaxation characteristics of three kinds of pork to establish prediction models of moisture content based on the LF‐NMR technology. According to the differential moisture‐binding strength of bound water, nonflowing water, and free water, the relaxation time can be recorded. The comparison of this relaxation time, in combination with the least‐squares method, partial least‐squares method, and principal component analysis method, verified the feasibility of low‐field nuclear magnetic resonance technology for the rapid detection of pork moisture content. Therefore, a quantitative analysis of the moisture content of migrating tilapia was undertaken to evaluate the effect of alternating magnetic fields on the moisture content of frozen tilapia after thawing (Cui et al., 2020).

Magnetic field‐assisted freezing has emerged as a new focus of national efforts. Tang's research group studied the influence of various magnetic field types and strengths on cherries during the freezing process. The cherries were exposed to a permanent magnetic field (PMF) ranging from 0–20 mT and an alternating magnetic field (AMF) ranging from 0–20 mT. The application of a magnetic field reduced the drip loss to a certain extent (Tang et al., 2020). Zhou Zipeng et al. investigated the effect of alternating magnetic fields on the supercooling process of water and physiological saline. The alternating magnetic field reduced the minimum noncrystallization temperature of the water and physiological saline. The increase in the degree of subcooling of the water formed a linear relationship with the magnetic field strength. Moreover, an increase in the subcooling degree of physiological saline formed a cubic polynomial relationship with the magnetic field strength (Zhou & Zipeng, 2013). Cui Ying et al. used Hetao melon to study the effect of alternating magnetic fields on the storage quality of these melons. The change in the quality was determined by controlling the treatment time of the magnetic field. The Hetao honeydew melons were treated with a constant magnetic field for 0, 5, 10, and 15 min, and their qualities were studied after storage at room temperature. After exposure to the magnetic field for 10 min, the appearance of the melons was better, the content of the soluble solids was relatively high, and there was little change in the content of the reducing sugars. The soluble protein content was high, the Vc loss was small, and the weight loss rate curve was relatively flat. These changes effectively extended the storage time of the Hetao melons, reduced the loss during postharvest storage, and improved its edible quality and commodity value. In order to improve pork muscle fibrinogen quality, low‐frequency alternating magnetic fields were used (Yang et al., 2020). It was observed that with the application of LF‐MF, the moisture ratio and mobility changed at different levels. LF‐MF improved bonding with water and, to varying degrees, altered alpha‐helix and β‐folding to modify the secondary structure of pork muscle fibrin. LF‐MF also drives molecular rearrangement and cross‐linking, causing the gel to capture more water and construct orderly networks. The influence of low‐frequency magnetic fields (LF‐MF; 0, 0.25, 0.5, and 1.4 MP) on the pork muscle fibrinogen (MP) gel properties (Wang, Xia et al., 2020) was studied. The treatment of 0.5 mT improved gel holding water capacity (WHC) and rheological properties much better compared to other processing. As the intensity increased, the fluidity of water did not change significantly, while the ratios of fixed water (PT21) and free water (PT22) were significantly reduced and increased, respectively. It shows that the effect of LF‐MF on MP hydration is related to the formation of water clusters, and it can affect its quality. In the work of Mohsendalvi‐Is Fahan, the final quality of frozen products depended to a large extent on ice crystal formation (Dalvi‐Isfahan et al., 2017). Since it causes irreversible damage to the microstructure of the food matrix, the application of magnetic field has a great research value in food. In summary, according to the existing practical experience of the alternating magnetic fields, researching its role in influencing the quality of frozen tilapia is of practical significance (Zhou & Zipeng, 2013).

Like all frozen meat products, the freezing quality of tilapia affects many factors. Among them, the formation of ice crystals during the freezing process, the loss of juice, and the destruction of the muscle tissue caused by the extrusion of the cell membranes are the important ones. The magnetic field can effectively interfere with the formation of ice crystals. At present, there are few studies on the quality improvement of frozen products by magnetic fields, and the improvement mechanism of frozen products is not very clear. In this experiment, a magnetic field was added during the freezing process of tilapia. The effects of the magnetic field‐assisted freezing were determined on the moisture content, water migration degree, microstructure, fractal dimension, etc., and the quality of the frozen tilapia. This study determined the magnetic field strength that could be useful in magnetic field‐assisted freezing for improving the quality of frozen tilapia.

## 2 MATERIALS AND METHODS

### 2.1 Construction of the experimental equipment

**FIGURE 1:**
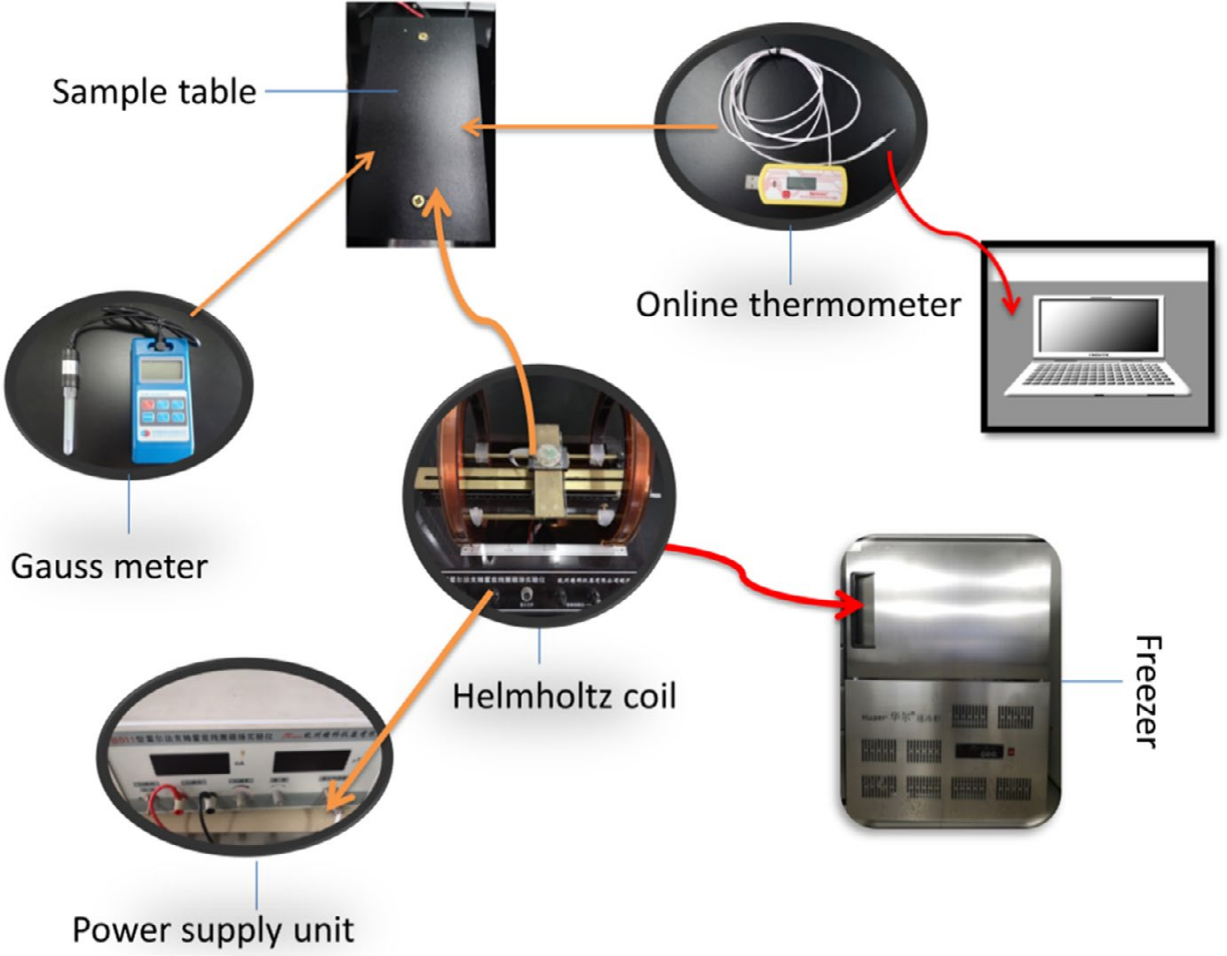
Diagram of the alternating magnetic field‐assisted freezing equipment

The experimental equipment was composed of the alternating magnetic field signal generator, the Helmholtz coil, and a low‐temperature quick‐freezer (Figure 1). The sample stage was set in the middle of the coil. The strength of the magnetic field was monitored regularly using a Gauss meter. After the signal generator was connected to the coil and placed in the quick‐freezer, tilapia samples on the sample stage were frozen by the magnetic field. The frequency of the signal generator was 50 Hz. An alternating magnetic field of 0 ~ 50 G was produced by changing the voltage and current values according to the needs of the experiment; the coil produced a constant magnetic field in a low‐temperature environment.

### 2.2 Sample processing

**FIGURE 2:**
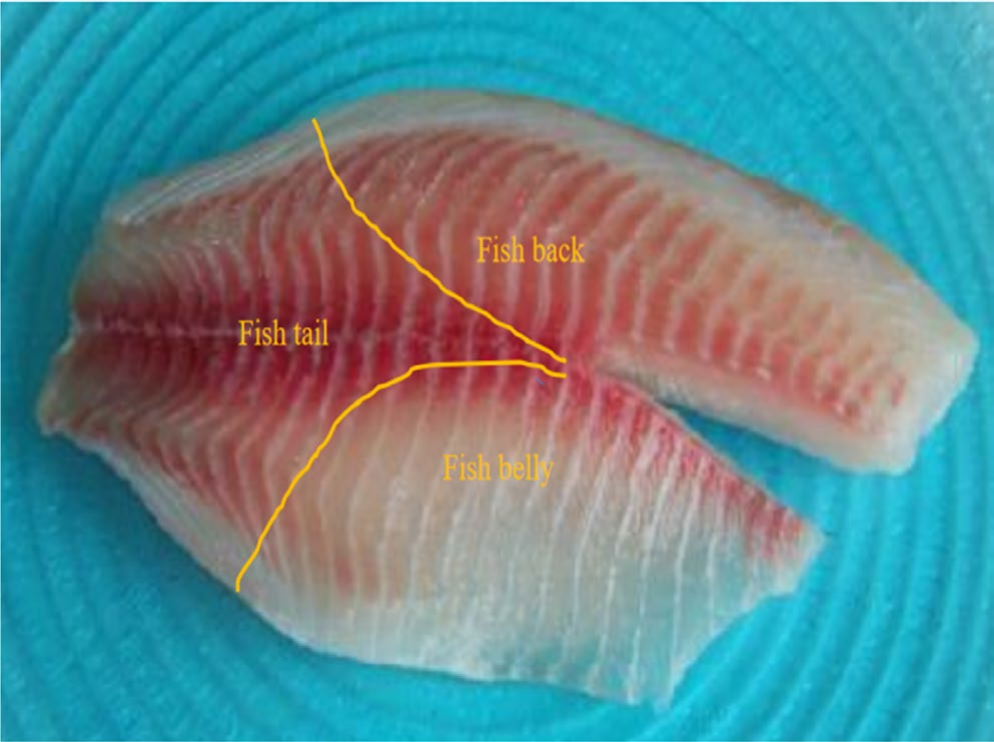
Zoning of the different parts of tilapia

Fresh tilapia samples weighing about 3/5 ounces were peeled, stabbed, the guts were removed, and they were divided into two pieces according to the requirements of the experiment (Figure 2). Thus, the tilapia fillets were divided according to the different regions of the abdomen, back, and tail (Figure 2). These parts were then exposed to alternating magnetic fields of strengths 10 G, 20 G, 30 G, 40 G, and 50 G in combination with a freezing treatment wherein the temperature of the freezer was –35°C. The control group was frozen in the absence of any magnetic field for the experiment.

### 2.3 Determination of water migration

The water migration reference method was modified slightly using the reference (Wang, Fu et al., 2020). A low‐field nuclear magnetic resonance instrument (NMI20–040H‐I, Shanghai Newman Co., Ltd.) was utilized for measurement. The processed samples of the fish's back, abdomen, and tail were cut into squares sized about 20 g. After absorbing the surface moisture of the samples, they were wrapped in plastic and inserted into the instrument. Next, the transverse relaxation time T2 of the sample was recorded through the Carr‐Purcell‐Meiboom‐Gill (CPMG) pulse sequence. During acquisition, the sampling frequency (SW) was set at 200 kHz, the resampling interval (TW) was 5,000 ms, the cumulative acquisition times (NS) were equal to 4, the echo time (TE) was 0.50 ms, and the number of echoes (NECH) was equal to 3,000.

After the CPMG test was completed, the sample was subjected to a multispin‐echo pulse sequence imaging test. The magnetic resonance imaging (MRI) imaging software and *MSE* multispin‐echo sequence were used to collect the coronal proton density image of the sample. During measurement, the repetition waiting time (TR) was set at 500 ms, the echo time (TE) was 18.125 ms, and the number of iterations was set at 100,000. The top view was chosen, and four levels were cut longitudinally for image acquisition. The collected images were uniformly mapped, filtered, and pseudo‐color processed, and then saved.

### 2.4 Determination of the volumetric moisture content

A Time Domain Reflectometer (Campbell Scientific TDR100, US) was combined with special software for the measurement. The instrument probe was inserted into the sample horizontally and perpendicular to the direction of the muscle texture of the sample. The insertion depth was 2 cm. The operating software selected the “volume moisture content” for measurement. The measurements of the abdomen, back, and tail samples of the fish were recorded thrice and the average was noted.

### 2.5 Frozen section and calculation of the fractal dimension and ice crystal area

Fish samples from different parts were selected. The back sample was cut into 2 × 2 × 2 cm pieces, and the tail and abdomen samples were cut into 2 × 2 × 1 cm pieces. The meat pieces were put into the cryostat after embedding them in the OCT (optimal cutting temperature) compound. The temperature of the microtome was –20°C, and the thickness of the slice was set to 10 µm. After the slicing was completed, the samples were placed on a glass slide for HE staining. Finally, the sample was observed with the Olympus BX41 optical microscope, and the picture was recorded (He et al., 2015).

The calculation of the fractal dimension was based on the picture of the freezer, and the entire binarized image was covered with a square subframe having a length of ε. The length of the square subframe was determined to be 1/2, 1/4, 1/8, 1/16, 1/32, and 1/64 of the side length of the image in turn. The number of grids covering the muscle tissue of the sample was recorded as *N*(1/ε), y was set at ln *N*(1/ε), x = ln 1/ε; the regression analysis was performed on x and y. Here, the slope of the straight line was the fractal dimension of the fish slice, and the ice crystal area was calculated by the “Summarize” function on the frozen slice image using the ImageJ software.

### 2.6 Quality determination

The sample was measured using the texture meter (CT3 Brookfield US). During measurement, the setting mode was TPA, the probe type was TA41, and the trigger point load was 7 g. Each sample was measured thrice in each measurement cycle, and the average value was recorded.

### 2.7 Fat oxidation value (TBA)

The method of Wang Chunling was used with slight modifications, 10.0 g of fish meat was weighed, 90 mL of 7.5% trichloroacetic acid (TCA) homogenate was mixed for 2 min (30 s, stop 30s), and extracted for 30 min. After 30 min, 5 mL of filtrate and 5 mL of thiovaleldic acid (TBA) (0.02 mol/L) were mixed and kept at 90°C in a water bath for 40 min. The mixture was cooled to room temperature, and then the absorbance A and TBA value (mg/kg) = 7.8 * a was measured at a wavelength of 532 nm. The malondialdehyde content was calculated from the TBA value.

### 2.8 Data statistics and analysis

Each set of samples was analyzed in duplicate, and the experimental data were the average of three experiments and expressed in the form of mean ±standard error. In the experiment, Origin 10, Microsoft PowerPoint, and ImageJ were used for drawing the graphs, and the SPSS 19.0 software was used for data processing and analysis.

## 3 ANALYSIS OF RESULTS

### 3.1 The effect of magnetic fields of different strengths on water migration in tilapia

**FIGURE 3:**
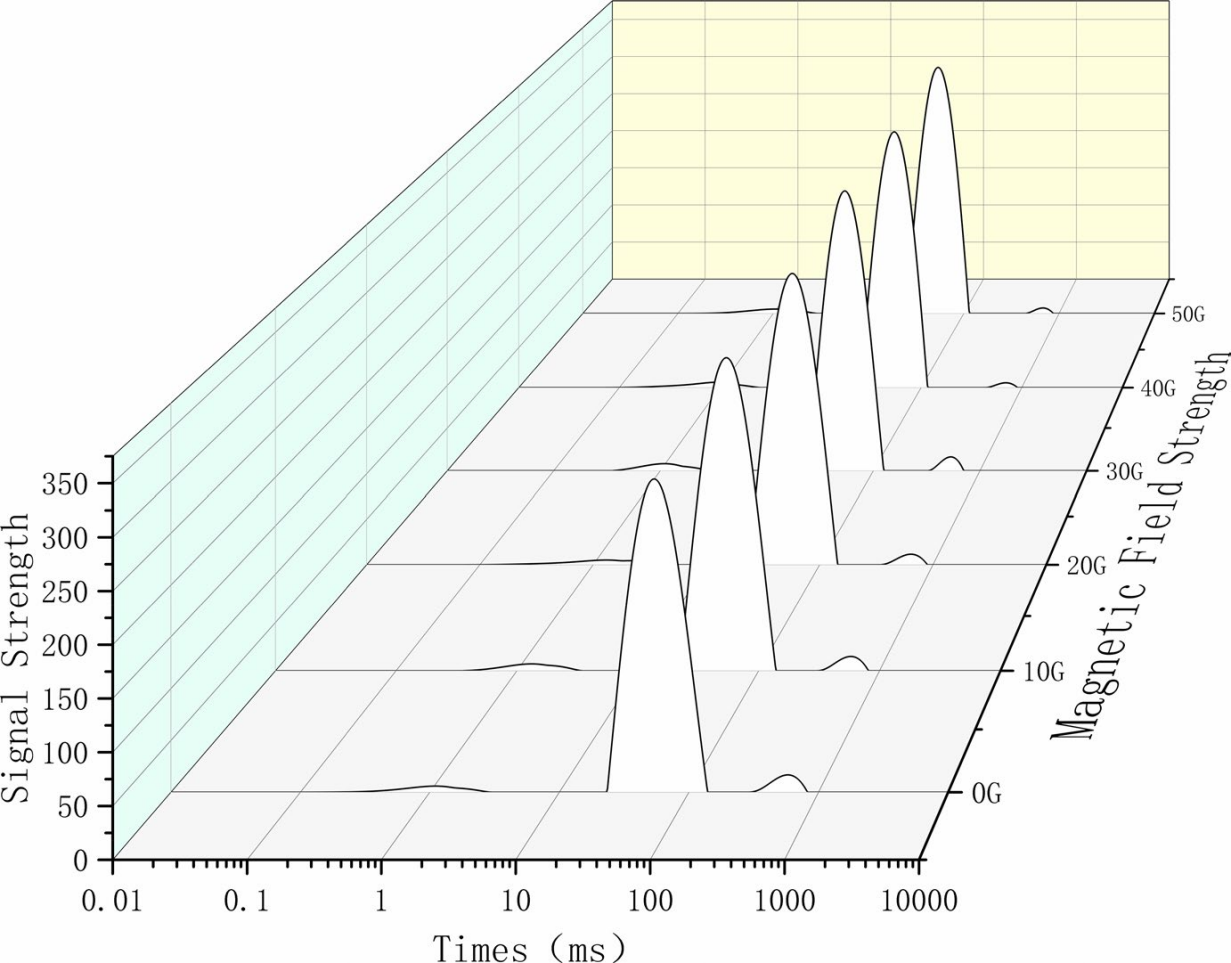
Moisturecontent in the back flesh of migrating tilapia under different strengths of magnetic fields

**FIGURE 4:**
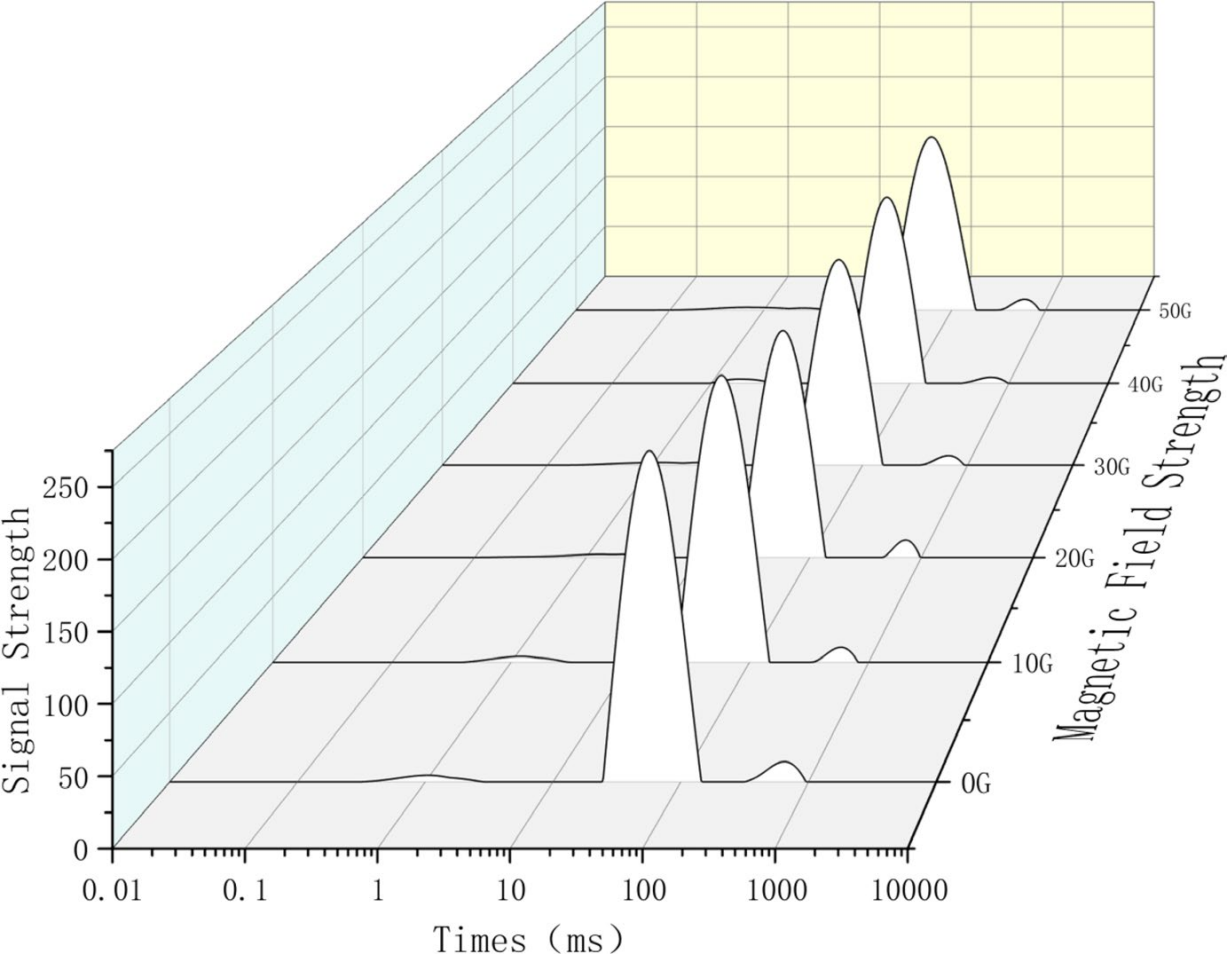
Moisture content in the abdomen flesh of migrating tilapia under different strengths of magnetic fields

**FIGURE 5:**
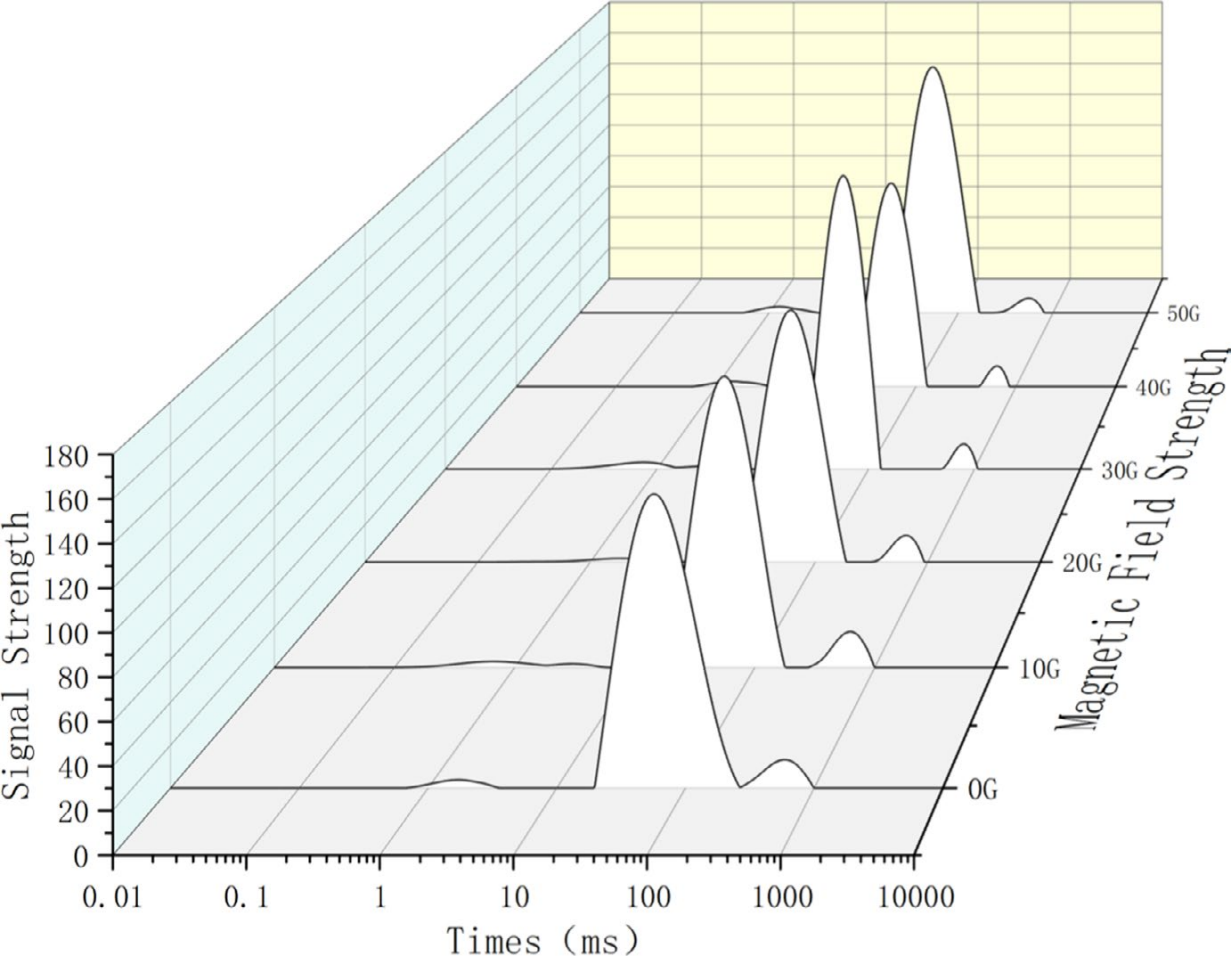
Moisture content in the tail flesh of migrating tilapia under different strengths of magnetic fields

The respective water distribution in the samples of the back, abdomen, and tail of tilapia under the influence of the magnetic fields of strength 0 ~ 50 G is shown in Figures 3, 4, 5. According to the principle of NMR relaxation, the peak appearing in 0.1 ~ 1 ms (T2b) represented strongly bound water, the peak appearing in 1–10 ms (T21) represented weakly bound water, the peak appearing in 10–100 ms (T22) represented water that was not easy to flow, and the peak appearing in 100 ~ 1,000 ms (T23) indicated free water. Based on the changes in the peak value and peak area, the water content and water migration of fish fillets treated with magnetic fields of different strengths were analyzed. Moreover, the effect of alternating magnetic field‐assisted freezing on the quality of tilapia was evaluated (Wang et al., 2019; Zhang et al., 2021). In this experiment, under freezing conditions and treatment with magnetic fields of different strengths, three peaks appeared in the back samples. The water migration diagram for the back samples (Figure 3) indicated that the control group shifted to the right‐most side, and the water mobility increased significantly; the free water content changed significantly. Compared with the control group, the water migration was similar in the groups exposed to magnetic intensities of 10 G and 20 G, and the effect of the magnetic field was not obvious. The effect of magnetic field‐assisted freezing on groups exposed to 40 G and 50 G of magnetic intensities was the most obvious. For the abdominal samples of tilapia (Figure 4), this effect was most obvious in the groups exposed to 30 G and 40 G of magnetic strength followed by those exposed to 50 G and 20 G strengths; these are the results in comparison with the control group. The water migration in these abdominal samples was significantly better than that of the control group after magnetic field treatment. For the tail samples (Figure 5), four kinds of peaks were visible in the water migration; that is, the bound water was divided into strong bound water and weak bound water at different times. This may be because the moisture in the tail of tilapia was not tightly bound. During the freezing process, low‐temperature freezing exerts a greater impact on the quality of the tail fish. Therefore, the application of magnetic field‐assisted freezing exerted a significant effect on water migration in the tail of tilapia. The water content of the control group shifted significantly to the right compared with that observed in the magnetic field‐treated groups.

The above analysis demonstrated that magnetic field‐assisted freezing promoted the quality of frozen tilapia. However, the effect of different strengths of magnetic fields was different in different parts, and there was no regular change. Among the groups, the effect of 40 G and 50 G magnetic field strength was the best on the back, and magnetic intensities of 40 G and 30 G were most effective on the abdomen and the tail. The differential effects may be due to the differential tightness of the tail, abdomen, and back regions of tilapia and the difference in the water contents of these body parts. However, a comprehensive analysis illustrated that the auxiliary freezing of tilapia in magnetic fields of strength 30 G, 40 G, and 50 G exhibited a significant promotional effect on their water migration compared with the control group.

### 3.2 The influence of differential magnetic fields on the T2 peak area of transverse relaxation

The transverse relaxation peak area is an effective response to the moisture content of various components. The moisture changes of bound water, nonflowing water, and free water can be directly observed through the peak area (Tang et al., 2019). According to the A23 peak area (Table 1), the peak areas of the back, abdomen, and tail samples displayed a decreasing trend when the magnetic field was gradually increased. The total peak area of the abdomen and tail exhibited a low increase in the group treated with a 50 G magnetic field. However, compared with the control group, all peak area values of A23 were lower than those of the control group. Moreover, there were significant differences in each part of the tilapia in the control group and the group treated with the magnetic field intensity of 40 G (*p* < .05). The 40 G group had the lowest peak area value of the back sample. This showed a significant difference compared to the control group (*p* < .05), but there was no difference compared to the 50 G group. There was no significant difference in the peak area values between the three groups (10 G, 20 G, and 30 G) (Liu et al., 2017). In the analysis of the peak area of A23 in this experiment, differences existed in the moisture content and meat quality between various parts of tilapia. When magnetic fields of 30 G, 40 G, and 50 G intensities were applied, the best effect was visible on the back fish meat, and a magnetic field of 30 G and 40 G displayed the best effects on the abdomen and back.

**TABLE 1:** Tilapia A23 peak area under different magnetic field strengths

Magnetic field strength	Fish back	Fish belly	Fish tail
0 G	42.67 ± 1.20^b^	57.00 ± 2.52^c^	90.00 ± 1.15^b^
10 G	41.00 ± 1.15^b^	50.67 ± 1.45^bc^	91.00 ± 2.52^b^
20 G	38.67 ± 1.86^b^	57.00 ± 1.53^c^	90.67 ± 1.20^b^
30 G	38.67 ± 1.45^b^	47.00 ± 1.54^ab^	82.00 ± 3.00^a^
40 G	29.67 ± 2.91^a^	41.67 ± 2.60^a^	81.67 ± 1.20^a^
50 G	30.00 ± 2.89^a^	53.33 ± 1.86^bc^	78.33 ± 1.21^a^

Note

Alphabets in the same column indicate significant differences.

Different superscript alphabets in the same column indicate significant difference (*p* < .05).

### 3.3 MRI images of differential magnetic strengths

**FIGURE 6:**
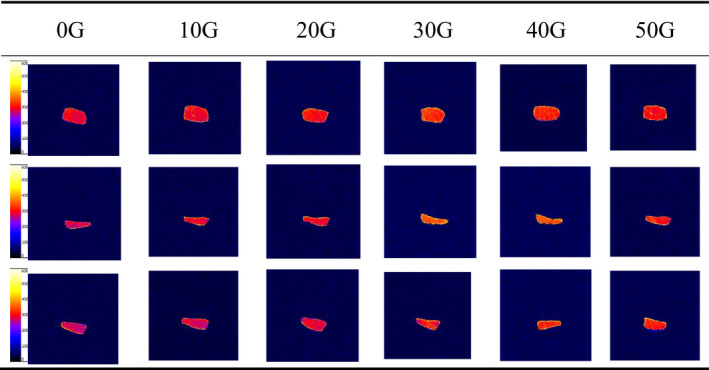
Magnetic resonance imaging images of tilapia under different magnetic field strengths

The pseudo‐color map of the different parts of tilapia obtained from the MRI analysis is depicted in Figure 6. The first three rows describe the pseudo‐color images obtained after exposure to magnetic fields of different strengths for the back, abdomen, and tail regions of the tilapia. The color, brightness, and saturation of the imaging were analyzed by comparing with the chart on the left. Generally, the closer the imaging color is to red or yellow, the stronger is the vividness, and the higher is the moisture content. In this experiment, the application of the magnetic field did not show a positive effect on the samples of the back meat when compared with the control group. However, the group treated with a magnetic strength of 10 G was an exception. The other groups displayed completely bright colors and exhibited good water holding capacity. The groups of abdominal meat samples treated with magnetic intensities of 30 G and 40 G displayed better water holding capacity. The groups of the tail samples treated with magnetic field intensities of 40 G and 50 G exhibited a better effect. Thus, a comprehensive analysis revealed that although the effect of different magnetic field intensities on different parts of the tilapia body was not regular, compared with the control group, the magnetic field‐assisted freezing exhibited a better effect on the water holding capacity of the frozen tilapia (Wei et al., 2021).

### 3.4 The effect of magnetic fields of different strengths on the volumetric water content in tilapia

The volumetric moisture content is an important indicator to effectively measure the quality of the frozen tilapia. In the freezing process of meat products, the freezing of moisture between the tissues can adversely affect the quality of the meat. The results of the different parts of tilapia were obtained by the time domain reflectometer (Table 2). Herein, the volume of water content in the back samples was higher than that of the control group under the influence of alternating magnetic field‐assisted freezing. The volumetric water content in decreasing order was 50 G > 40 G > 20 G > 30 G > 10 G > 0 G; the 50 G and 40 G groups were significantly different from the control group (*p* < .05). The order of the volumetric water content in the abdominal flesh in decreasing order was 30 G > 50 G > 40 G > 10 G > 20 G > 0 G; only the 40 G and 50 G groups displayed significant differences when compared with the control group (*p* < .05). The volumetric water content of the fish in the tail samples did not show a gradual increase with an increase in the magnetic field; however, the changes were irregular. The reason may lie in the strength of the magnetic field applied during the freezing process. This strong magnetic field may interfere with the formation of ice crystals, thereby reducing their size and also reducing the damage to the muscle tissues and the loss of juice. However, due to differences in the water holding capacities between the muscles, the effect was different. As the magnetic field increased, the volume of the water content of the tail samples appeared to reduce the loss of juice. This may be because the tail of the fish had less water content and froze more rapidly during magnetic field‐assisted freezing; hence, the effect of the magnetic field was more obvious. Generally speaking, the effect of the magnetic field on the frozen tilapia was positively correlated (Lou et al., 2013).

**TABLE 2:** Volumetric water content under magnetic fields of different strengths in tilapia

Magnetic field strength	Fish back (%)	Fish belly (%)	Fish tail (%)
0 G	73.83 ± 0.54^a^	70.57 ± 0.71^a^	49.80 ± 0.42^a^
10 G	75.23 ± 0.52^a^	71.27 ± 0.73^a^	50.97 ± 0.71^ab^
20 G	75.10 ± 0.64^a^	71.23 ± 0.82^a^	51.70 ± 0.82^ab^
30 G	75.83 ± 0.21^a^	72.80 ± 0.47^b^	52.97 ± 0.58^bc^
40 G	76.17 ± 1.11^b^	72.20 ± 0.32^a^	54.23 ± 0.63^c^
50 G	76.80 ± 0.60^b^	72.80 ± 0.62^b^	54.23 ± 0.77^c^

Note

Alphabets in the same column indicate significant differences.

Different superscript alphabets in the same column indicate significant difference (*p* < .05).

### 3.5 Microstructure analysis of magnetic fields of different strengths

**FIGURE 7:**
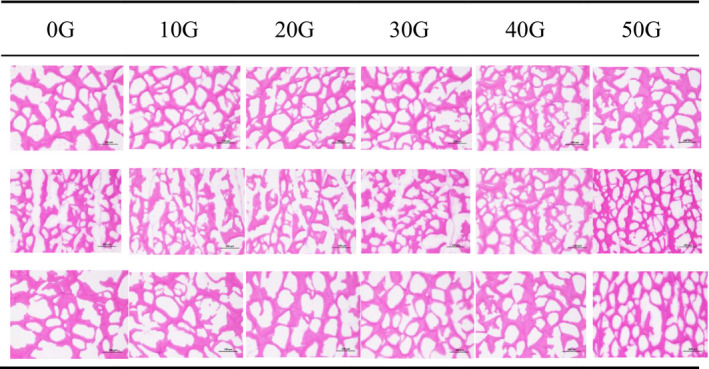
Frozen sections of tilapia under magnetic fields of different strengths

The fundamental reason for the improvement in the quality of frozen products by magnetic field‐assisted freezing is the reduction in the volume of ice crystals and the formation of small and many crystal clusters during the freezing process (Jiang et al., 2019b); this also reduces the damage between the muscle tissues caused by freezing (Shi et al., 2019). In the microstructure of the frozen section (Figure 7), the stained part represents the muscle tissue, and the blank part indicates the ice crystal area left by the sublimation of water after alcohol rinsing. The picture was magnified 200 times. The first three rows depict the microstructure diagrams of the back, abdomen, and tail samples, respectively. For the samples of the back region, the effect of magnetic intensities of strengths 10 G, 20 G, and 30 G was not obvious in comparison with that of the control group. Compared with the control group, the ice crystals formed in the groups treated with magnetic intensities of 40 G and 50 G groups displayed a significant decreasing trend. The number of irregular ice crystals in the abdominal fish samples significantly decreased with an increase in the magnetic field when compared with the control group. This was especially true in the group treated with the magnetic field intensity of 50 G and 40 G, wherein ice crystals of regular shape were formed. For the tail samples, as the magnetic field strength increased during freezing, the ice crystal size gradually decreased. This result was consistent with the analysis of the volumetric water content. Through microstructure analysis, in addition to the 10 G magnetic field, differential effects were observed in different parts of tilapia due to differences in the water content and muscle tissue. Although the effects were different, in general, magnetic field‐assisted freezing significantly promoted the formation of ice crystals during the freezing process of tilapia, and consequently, helped to improve the quality of frozen tilapia (Jiang et al., 2019a).

### 3.6 The effect of magnetic fields of different strengths on fractal dimension and ice crystal area ratio

The fractal dimension reflects the effectiveness of complex shapes occupying space. It is a measure of the irregularities of complex shapes. By analyzing the complexity of the two‐dimensional images of frozen sections, the fineness of the ice crystals can be visualized through the fractal dimension. The smaller the ice crystals are the more and the higher is the complexity, and the higher is the fractal dimension value. The calculation of the ice crystal area ratio effectively measures the ratio of ice crystals to muscle tissue after freezing and thawing. The smaller the ice crystal area ratio (size of the ice crystals), the lower is the deformation between the tissues of the sample after thawing. The fractal dimension and the ice crystal area of the frozen section image were calculated using ImageJ software (Li et al., 2020).

**FIGURE 8:**
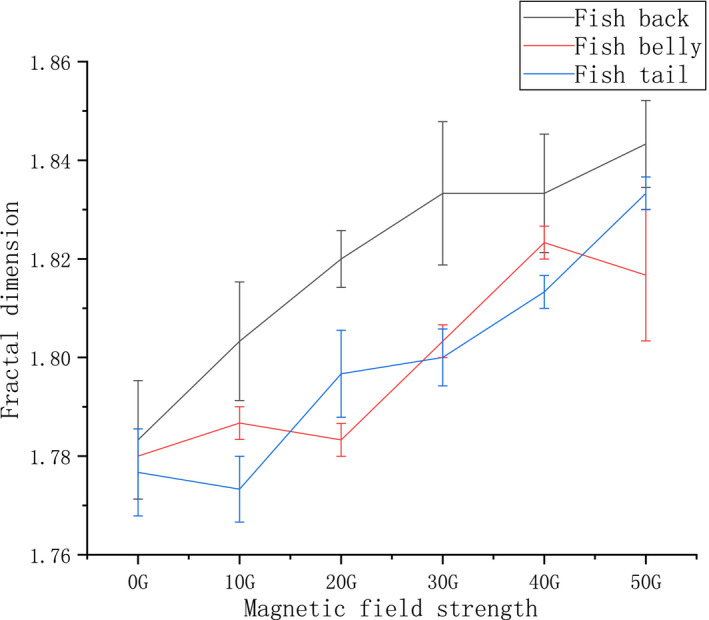
Fractal dimension changes under magnetic fields of different strengths in tilapia

The fractal dimension analysis depicting the influence of magnetic field‐assisted freezing on different parts of tilapia is shown in Figure 8. For the back samples, the fractal dimension value in decreasing order was 50 G > 40 G = 30 G > 20 G > 10 G > 0 G. In addition to 10 G, the other groups also displayed significant differences when compared with the control group (*p* < .05). The fractal dimension value of the abdominal samples in decreasing order was 40 G > 50 G > 30 G > 10 G > 20 G > 0 G. Significant differences were observed in the groups treated with the magnetic intensities of 30 G, 40 G, and 50 G when compared with the control group. The fractal dimension value of the tail sample in decreasing order was 50 G > 40 G > 30 G > 20 G > 0 G > 10 G. The poor performance of the 10 G group in comparison with the control group may be because the fractal dimension value reflects the complexity of the muscle tissue. The formation of ice crystals during the freezing process expands the volume of the sample and results in the squeezing and deformation of the intermuscular tissue. The auxiliary freezing of different magnetic field strengths has different effects on the formation of ice crystal clusters. The change is not regular, so this situation occurs.

**FIGURE 9:**
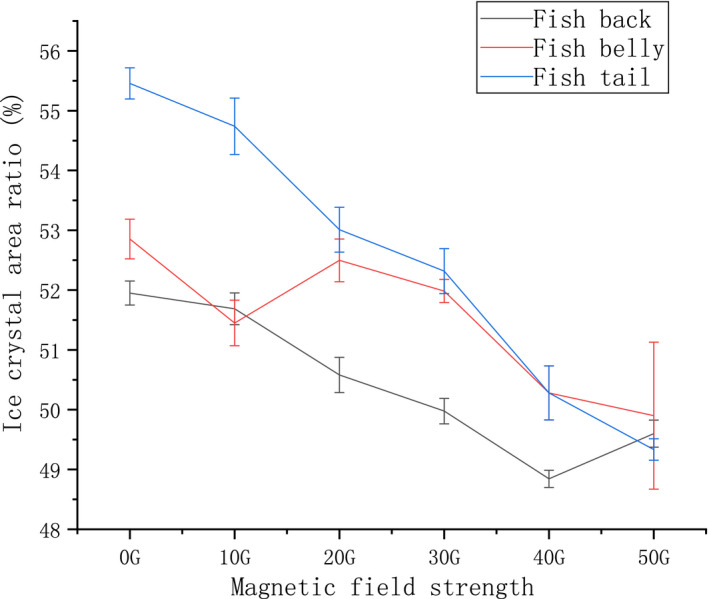
Changes in the area ratio of tilapia ice crystals under magnetic fields of different strengths

An analysis diagram of the ice crystal area ratio of samples at different positions under different magnetic field strengths is shown in Figure 9. The ice crystal area ratio of each part of the tilapia sample decreased in comparison with the control group in the presence of a magnetic field. Upon increasing the strength of the magnetic field on the back samples, the ice crystal area ratio increased in the group treated with 50 G of magnetic field strength; the ice crystal area ratio of the other groups showed a negative correlation when compared with the control group (Cai et al., 2020). This may be due to the differential effects of the magnetic fields. Within the scope of this experiment, 40 G was the extreme point for the samples of the back fish meat. The abdominal fish samples treated with 10 G of magnetic strength exhibited a considerable decrease compared with the control group although the difference was not significant enough (*p* > .1). Compared with the control group, the 40 G and 50 G groups were significantly different (*p* < .05). For the tail samples, the ice crystal area ratio gradually decreased with an increase in the magnetic field strength; thus, a significant negative correlation was observed.

### 3.7 Effects of different magnetic field strengths on the texture of tilapia

The texture is an important indicator of the quality of the refrigeration product. It can effectively analyze the quality of the frozen nonfish through the formation analysis. Hardness reflects the force required by the sample to reach a certain amount, elasticity reflects the degree of recovery of the sample to reach a variation and reflects the relative resistance after the sample is compressed (Bedane et al., 2018). As shown in Table 3, it is the texture data that is frozen in magnetic fields of different strengths. A differential degree of impact was observed on the back, abdomen, and tail regions of the fish when the magnetic field was applied. Among these, the flexibility and hardness of the back samples were significantly different in the groups treated with magnetic fields of intensities 30 G, 40 G, and 50 G from the control group. The cohesiveness in the group treated with 40 G of magnetic field intensity was significantly different from that of the control group and the group treated with 50 G of magnetic field intensity (*p* < .05). For the abdominal fish samples, no significant differences were observed in the groups treated with magnetic field intensities 10 G and 20 G with an increase in the magnetic field strength. The hardness of the tail samples reached an extreme point in the group treated with a magnetic intensity of 40 G. This was the best effect on the elasticity and cohesiveness wherein the groups treated with 40 G and 50 G displayed significant differences compared to other groups (*p* < .05). The degree of hardness between the tail, abdomen, and back was huge because of the differences in the muscle thickness of the different parts. Moreover, the measurement distance set was different, so the data difference was obvious. (Jiang et al., 2019a).

**TABLE 3:** Effect of magnetic fields of different strengths on tilapia

	Magnetic field strength	Hardness (g)	Elasticity (mm)	Chewiness (g)
Fish back	0 G	327 ± 5.4^a^	2.74 ± 0.48^a^	0.47 ± 0.01^a^
	10 G	324 ± 5.1^a^	2.86 ± 0.34^ab^	0.49 ± 0.01^ab^
	20 G	328 ± 5.0^a^	2.97 ± 0.35^bc^	0.5 ± 0.02a^b^
	30 G	299 ± 7.5^b^	2.97 ± 0.49^bc^	0.5 ± 0.02^ab^
	40 G	289 ± 4.9^b^	3.04 ± 0.47^c^	0.52 ± 0.01^bc^
	50 G	293 ± 3.9^b^	3.01 ± 0.4^c^	0.55 ± 0.01^c^
Fish belly	0 G	195 ± 3^c^	1.47 ± 0.02^a^	0.58 ± 0.02^a^
	10 G	194 ± 3^c^	1.52 ± 0.01^ab^	0.57 ± 0.02^a^
	20 G	189 ± 2^bc^	1.49 ± 0.03^ab^	0.61 ± 0.03^ab^
	30 G	186 ± 2^ab^	1.53 ± 0.02^b^	0.63 ± 0.01^b^
	40 G	181 ± 1^a^	1.59 ± 0.01^c^	0.64 ± 0.01^b^
	50 G	180 ± 4^a^	1.61 ± 0.01^c^	0.66 ± 0.02^b^
Fish tail	0 G	204 ± 5^c^	1.48 ± 0.02^a^	0.58 ± 0.02^a^
	10 G	203 ± 3^c^	1.52 ± 0.02^ab^	0.59 ± 0.02^ab^
	20 G	198 ± 3^c^	1.51 ± 0.02^ab^	0.61 ± 0.01^abc^
	30 G	196 ± 3^bc^	1.54 ± 0.01^b^	0.62 ± 0.01^bc^
	40 G	188 ± 5^a^	1.6 ± 0.02^c^	0.64 ± 0.01^c^
	50 G	190 ± 2^ab^	1.62 ± 0.01^c^	0.63 ± 0.02^c^

Note

Alphabets in the same column indicate significant differences.

Different superscript alphabets in the same column indicate significant difference (*p* < 0.05).

### 3.8 Effects of different magnetic field strengths on the TBA value of tilapia

**FIGURE 10:**
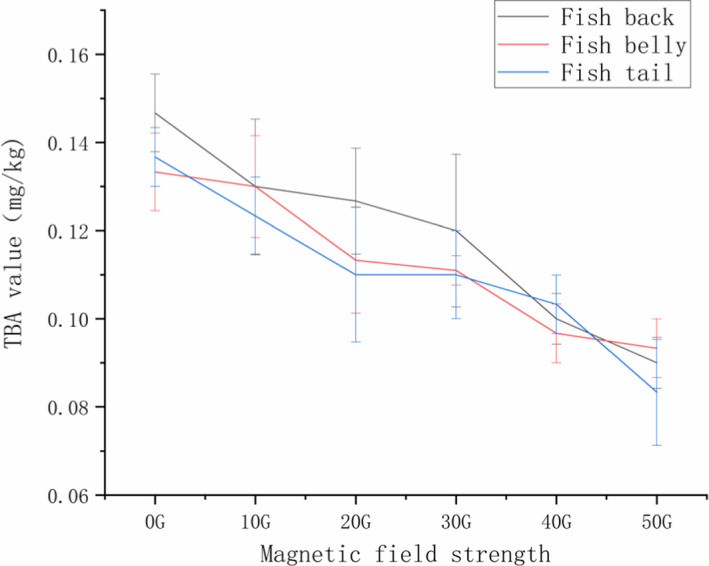
Tilapia TBA values under different magnetic field strengths

The loss of quality caused by fat oxidation is very important in food technology (Cheng et al., 2015). Peroxides degrade fats and produce secondary products such as alolytic and ketonic compounds. This produces a pungent smell and seriously affects the sensory and other qualities of frozen meat products. As can be seen from Figure 10, the alternating magnetic field applied during the process of freezing can effectively reduce the TBA value. For the tilapia back sample, as the magnetic field strength increases, there is a significant decrease in TBA value in 40 G and 50 G groups, which is 0.1 ± 0.00577, 0.09 ± 0.00577, respectively. These values are significantly different from the control group (*p* < .05). The abdominal sample showed a gradual decline in TBA value, but there is no significant difference between 20 G and 30 G, and 40 G and 50 G groups (*p* > .05). The tail sample showed no significant difference between 20 G group and the control group. The preservative effect of the magnetic field on fat oxidation is clear. This could be due to the fact that the addition of a magnetic field during freezing reduces water crystallization in the cell membrane and improves the oxidation resistance of tilapia.

## 4 CONCLUSION

The tilapia samples of the back, abdomen, and tail regions illustrated improved quality compared to the control group after exposure to alternating magnetic fields of different intensities. For the groups treated with magnetic fields of 40 G and 50 G, the moisture content reduced significantly, the free water content decreased, the fractal dimension value increased, the crystal area reduced, and the refreshing damage between the muscle tissue was reduced. The hardness and elasticity also were significantly different from those in the control group, and the quality had significantly improved (*p* < .05). The moisture content in the abdominal samples treated with magnetic fields of 30 G, 40 G, and 50 G was reduced. Microstructure and ice crystal area ratio analysis indicated a reduced crystal area and crystal size when compared with the control group; the texture indicator exhibited the same significant difference with the control group (*p* < .05), TBA value decreased. The moisture migration was preferable in the groups treated with magnetic fields of 30 G and 40 G, and the fractal dimension and ice crystal area ratio were preferable in the group treated with 50 G of the magnetic field. The groups treated with magnetic intensities of 10 G and 20 G did not exhibit significant differences when compared with the control group (*p* > .05). The hardness was best in the group treated with the magnetic field of 40 G intensity. Therefore, after a comprehensive analysis, it was concluded that an alternating magnetic field and auxiliary freezing exerted a promotional effect on the quality of tilapia. Among the different magnetic intensities tested, the groups treated with 30 G, 40 G, and 50 G showed the best results. Thus, the study provided a theoretical reference for the utility of magnetic fields in the storage preservation of tilapia. This study also provides a new way to use the magnetic field for the cold storage of seafood.

## CONFLICT OF INTEREST

We declare that we have no financial and personal relationships with other people or organizations that can inappropriately influence our work, there is no professional or other personal interest of any nature or kind in any product, service and/or company that could be construed as influencing the position presented in, or the review of, the manuscript entitled.

## ETHICAL APPROVAL

This article does not contain any research conducted by any author on animals or humans, and the informed consent of all individual participants in the research has been obtained. In addition, all procedures performed in the research are in compliance with the ethical standards of the institution and/or the National Research Council, as well as the 1964 Helsinki Declaration and subsequent amendments or similar ethical standards.

## Data Availability

The [DATA TYPE] data used to support the findings of this study are included within the article, The [DATA TYPE] data used to support the findings of this study are available from the corresponding author upon request.
